# Correction: Díaz-Talavera et al. PrimPol: A Breakthrough among DNA Replication Enzymes and a Potential New Target for Cancer Therapy. *Biomolecules* 2022, *12*, 248

**DOI:** 10.3390/biom12050693

**Published:** 2022-05-12

**Authors:** Alberto Díaz-Talavera, Cristina Montero-Conde, Luis Javier Leandro-García, Mercedes Robledo

**Affiliations:** 1 Hereditary Endocrine Cancer Group, Human Cancer Genetics Program, Spanish National Cancer Research Centre (CNIO), 28029 Madrid, Spain; cmontero@cnio.es (C.M.-C.); ljleandro@cnio.es (L.J.L.-G.); mrobledo@cnio.es (M.R.); 2 Centro de Investigación Biomédica en Red de Enfermedades Raras (CIBERER), Instituto de Salud Carlos III (ISCIII), 28029 Madrid, Spain

To improve the quality of the original article [1], some changes were made to the references. Two references were relocated: reference 98 in the original article is now reference 15 [2] and reference 133 in the original article is now reference 14 [3]. Minor modifications were made to the original text and figures to cite exclusively peer-reviewed research articles. These amendments are detailed below.

The specific changes included in the updated version are listed below. The amended text is underlined:

**Abstract.** In contrast with conventional primases, PrimPol is a DNA primase able to initiate DNA synthesis de novo using deoxynucleotides, discriminating against ribonucleotides. In vitro, PrimPol can act as a DNA primase, elongating primers that PrimPol itself sythesizes, or as translesion synthesis (TLS) DNA polymerase, elongating pre-existing primers across lesions. However, the lack of evidence for PrimPol polymerase activity in vivo suggests that PrimPol only acts as a DNA primase.

**Figure 3A.** Asterisks representing lesions were shifted to the left to represent primase activity of PrimPol only beyond the lesion. Figure 3 should be corrected as below.

**Section 2.3 (text related to Figure 3A).**However, the PrimPol ability to prime downstream of a lesion has only been demonstrated in vivo, which makes this enzyme the first DNA primase with this function to be discovered [13]. PrimPol is able to catalyze the synthesis of de novo primer downstream of a readable lesion or an unreadable lesion (Figure 3A). Considering the in vitro abilities of PrimPol, it is probable that in vivo PrimPol uses its abilities to be proficient as a DNA primase involved in restarting stalled replication forks. In summary, PrimPol is a primase able to catalyze long DNA primers ahead of lesions with its regular DNA primase activity. Yet, only in vitro, PrimPol is able to use its TLS capability when polymerizing in two different ways: on the one hand, by directly reading lesions, such as 8oxodG; and, on the other hand, by skipping non-readable lesions, such as AP sites, realigning the nascent primer ahead to continue the synthesis (Figure 3B). Traditionally, it has been speculated that the reason why PrimPol catalyzes the synthesis of DNA primers could be easily explained by the fact that DNA primers are suitable substrates for replicative DNA polymerases [74]. The fact that RNA primers catalyzed by RNA primases are easily eliminated, minimizing mutagenesis during replication, while DNA primers catalyzed by PrimPol, could be more difficult to be erased is also a matter of discussion [74]. Thus, it has been suggested that this special feature of PrimPol is required for its TLS polymerase activity [74].

**Table 1.** It was modified to reflect only critical residues for PrimPol function that have been described in peer-reviewed research articles. The corresponding references are included in the table. The legend of Table 1 was modified accordingly.

**Section 4 (text related to Table 1)**. In fact, specific mutations found in tumors, such as R76H and R76C mutations, likely impair PrimPol activity, as it has been previously shown for the Y100H mutation, which disables the entrance of dNTPs in favor of NTPs [74].

**Section 7 (text related to Table 1).** However, there are cancer types with a PrimPol CN loss (Figure 11D) or likely a loss of function mutations, such as those at residue R76 (Table 1). In fact, this residue is a mutation hotspot (Figure 11E). PrimPol alterations in tumors could mimic some features of the PrimPol-deficient phenotype observed in cell models: increased replicative stress and genomic instability compared to WT cells [13,94] (Figure 12D). Therefore, it is tempting to suggest that these patients could benefit from radiotherapy or chemotherapy treatments to which PrimPol-deficient cells have already been shown to exhibit hypersensitivity, such as the interstrand crosslink agents cisplatin [96,102,103] and mitomycin C [102], or even the combined treatment of camptothecin (CPT) or etoposide with olaparib.

**Figure 7A,B.** These figure panels were slightly modified to reflect only critical residues for the PrimPol function that have been described in peer-reviewed research articles. The deleted residues were also removed from the figure legend.

**Section 8.** Moreover, in vitro studies uncovered that PrimPol is able to use its TLS abilities when polymerizing [12,13], acting as a TLS DNA polymerase. In vivo, PrimPol DNA primase activity has been demonstrated; however, PrimPol TLS DNA polymerase action has not yet been probed.

**Addition of Acknowledgment**

## Figures and Tables

**Figure 3:**
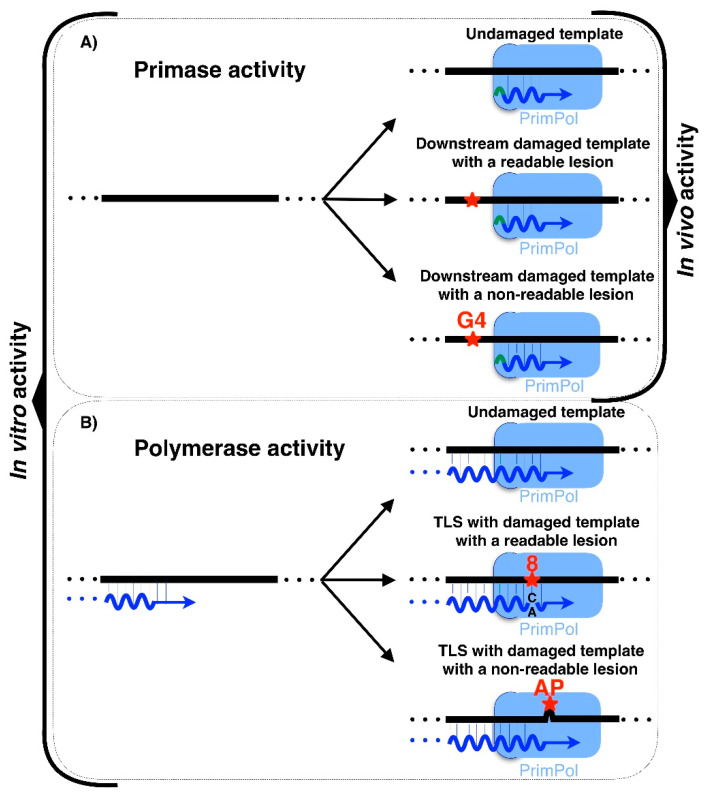
In vitro and in vivo activities of the human PrimPol. (**A**) Primase activity of PrimPol. (**B**) Polymerase activity of PrimPol. Both panels are broken down into polymerization with undamaged templates or damaged templates with both readable and non-readable lesions. Only the primase activity of PrimPol was shown both in vitro and in vivo. Polymerase activities are shown only in vitro and their relevance in vivo remains unclear. dNMPs are colored in blue, NTPs in green and DNA lesions in red. G-quadruplex are depicted as G4 in red, the 8oxodG lesion is depicted as an 8 colored in red (readable lesion), and the abasic site is depicted as AP colored in red (non-readable lesion). Asterisks indicate lesions.

**Figure 7:**
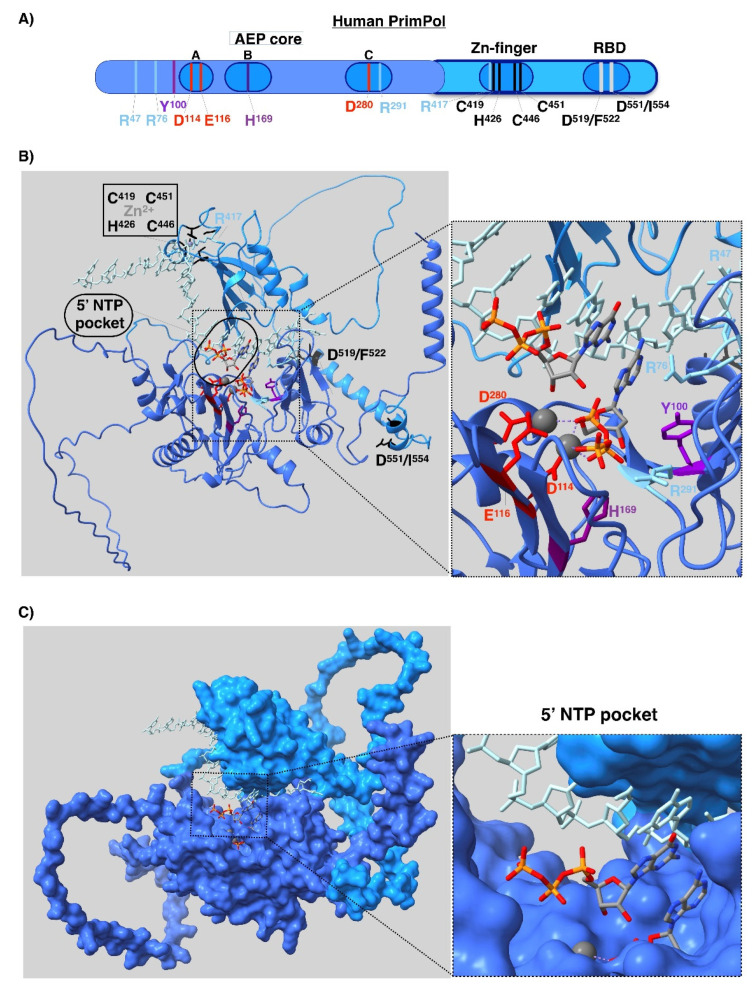
Structure of human PrimPol. (**A**) Linear representation of the structure. AEP domain is colored in blue and C-terminal region in light blue. In motifs (**A**–**C**) (which contain the carboxylates used to coordinate the metals and the key histidine to stabilize the incoming nucleotide), the Zn finger and the RPA binding domain (RBD) are highlighted. (**B**) Model structure obtained from AlphaFold v2.0 protein structure database and modeled in complex with DNA template and 3′dNTP by software fitting with PDB ID 7JKP, and 5′NTP by manual fitting with PDB ID: 7JKP. AEP domain is colored in blue and C-terminal region in light blue, which contains the Zn-finger domain and RPA binding sites, which are colored in black. The template is colored in orange, and 5′NTP and 3′dNTP are colored with CPK code. The key residues are shown in sticks: D^114^, E^116^, and D^280^ colored in red, H^169^ in purple, Y^100^ in dark purple, and R^47^, R^76^, R^291^, and R^417^ in blue. (**C**) Surface representation of the PrimPol structure model showing in detail the 5′NTP pocket.

**Table 1:** “Key residues of human PrimPol. Function, activity and cancer-related mutations compiled in COSMIC or GDC databases are indicated. Residues frequently mutated in cancer are indicated by an asterisk. The AEP domain is colored in blue and C-terminal region in light blue.”.

Residue	Function	Activity Involved [Ref.]	Somatic Mutations in Cancer (COSMIC Database)
R^47^	Contact the DNA template	Primase/polymerase [107,112]	-
*R^76^	Contact the DNA template	Primase/polymerase [107,112]	R76H and R76C
Y^100^	Steric gate (sugar selector)	Primase/polymerase [74]	Y100H
D^114^	Cation ligand (Mn^2+^ or Mg^2+^)	Primase/polymerase [12,79,107]	-
E^116^	Cation ligand (Mn^2+^ or Mg^2+^)	Primase/polymerase [12,79,107]	-
H^169^	Stabilize the 3′ nucleotide	Primase/polymerase [12,107]	-
L^200^-S^260^	PolDIP2 binding	Primase?/polymerase [116]	G201D, E203K, D204G, A208S, A208T, H214Y, P217S, P217L, H218Y, F219L, S220L, Q226L, K232T, M233I, T235R, W243S, T244A, G254W, SS59R
D^280^	Cation ligand	Primase/polymerase [12,79,107]	-
R^291^	Stabilize the 3′ nucleotide	Primase/polymerase [107,111]	R291W
*R^417^	-	-	R417L, R417W and R417Q
C^419^, H^426^,C^446^ and C^451^	Zn^2+^ ligand	Primase [12,113]	H426N and H426R
D^519^/F^522^ and D^551^/I^554^	Binding of RPA	Primase/polymerase [117]	F522V
